# Novel Epigenetic CREB-miR-630 Signaling Axis Regulates Radiosensitivity in Colorectal Cancer

**DOI:** 10.1371/journal.pone.0133870

**Published:** 2015-08-11

**Authors:** Yan Zhang, Jiang Yu, Hao Liu, Wenhui Ma, Li Yan, Jiefu Wang, Guoxin Li

**Affiliations:** Department of General Surgery, Nanfang Hospital, Southern Medical University, No. 1838 Guangzhou Avenue North, Guangzhou, 510515, Guangdong, China; University of Saarland Medical School, GERMANY

## Abstract

**Background:**

miR-630 has been reported to be a modulator of several cancers, but the mechanism by which is it influences radioresistance remains unknown. We aimed to identify the molecular function of miR-630 and its regulatory mechanism in colorectal cancer (CRC) cell lines.

**Methodology:**

Overexpression and loss-of-function analyses of miR-630 were performed in CRC cell lines by measuring their levels of growth and apoptosis after ionic radiation (IR). Target genes were detected via a dual-luciferase assay and Western blot. Chromatin immunoprecipitation assay was carried out to identify the transcription factor regulating miR-630, and a demethylation experiment was also conducted.

**Results:**

miR-630 expression was found to be positively correlated with radiosensitivity in CRC cell lines (*p*<0.05). After IR treatment, miR-630 induced apoptosis in cells; however, the opposite was observed when miR-630 was downregulated (*p*<0.05). BCL2L2 and TP53RK were identified as the target genes of miR-630, and the function of miR-630 was found to depend on these two genes (*p*<0.05). In addition, evidence showed that CREB regulates the level of miR-630, and demethylation can elevate miR-630 levels (*p*<0.05).

**Conclusion:**

CREB–miR-630–BCL2L2 and TP53RK comprise a novel signaling cascade regulating radiosensitivity in CRC cell lines by inducing cell apoptosis and death.

## Introduction

Colorectal cancer (CRC) is a relatively common type of cancer with high worldwide mortality rate [1, 2]. Although preoperative treatment with chemoradiotherapy (CRT) in combination with conventional surgery improves local control of CRC and survival, only about 70% of patients respond to CRT [3, 4]. Thus, understanding the mechanisms involved in ionic radiation (IR) resistance of CRC is an important step in generating further effective therapies.

MicroRNAs are small (18–25 nucleotides) noncoding RNAs that can suppress mRNA expression by binding to their complementary sequences in 3′ untranslated regions (UTRs) [5, 6]. Many studies have demonstrated that miRNAs play important roles in various biological processes [7]. Some miRNAs such as miR-135 and miR-200c have been found to play essential roles in radioresistance [8, 9]. A previous study reported miR-630 as a novel modulator of cisplatin- induced non-small-cell lung cancer cell death [10]. Microarray analysis in a clinical research selected a set of 13 miRNAs, including miR- 630, which may be specific predictors of CRT outcome in rectal cancer patients [11]. However, the mechanism by which miR-630 influences radioresistance has not been elucidated to date. In the current study, we sought to identify the function of miR-630 in CRC radiosensitivity and its potential regulator.

## Materials and Methods

### Cell culture

Human colorectal cancer cell lines Ls174T, SW480, HCT116, SW837, HR8348, and HT29 were purchased from the Cell Bank of Type Culture Collection (Shanghai City, China). All of these cell lines were maintained in RPMI 1640 medium containing 10% fetal bovine serum (HyClone, Logan, Utah, USA) in a 37°C humidified incubator under 5% CO2.

### MiRNA extraction and quantitative real-time PCR (qRT-PCR)

TRIzol reagent (Invitrogen, Foster City, USA) was used to isolate total RNA from cultured cells. qRT-PCR was performed on a 7500 System (Applied Biosystems, Foster City, USA) using an all-in-One miRNA Reverse Transcription Kit (GeneCopoeia, Inc.) and SYBR Green Human miRNA Assay Kit (GeneCopoeia, Inc.).

### Cell irradiation

Irradiation was performed in a Siemens Meratron M2 medical irradiator at a dose of 3 Gy at room temperature.

### Cell proliferation assay

In 96-well plates, 1×10^3^ cells were seeded and then incubated for 4 d. Cell counting kit-8 (CCK-8)(KeyGene BioTECH) assay was performed to measure cell proliferation. Briefly10 μL of CCK-8 solution was added into each well for 2 hours. The absorbance of each well was read using a microplate reader set at 450 nm.

### Caspase 3/6 activity assay

Caspase 3/6 activity was measured using Caspase 3 and Caspase 6 assay kits (Abcam, USA). Cell lysis buffer was added at a volume of 50 μL to resuspend 1×10^6^ cells and incubate the cells on ice for 10 min. Then, DEVD-p-NA substrate (or VEID-p-NA) and reaction buffer containing DTT was added, and the mixture was incubated for 2 hours at 37°C. Subsequently, the samples were read at 450 nm using a microtiter plate reader.

### Apoptosis assay

Cells were collected 24 hours after radiation with or without 3 Gy IR. Cells were stained with Annexin V-FITC (KeyGene BioTECH) and PI (KeyGene BioTECH). Apoptosis was performed using flow cytometry (BD LSRFortessa).

### Dual-luciferase assay and vector construction

TargetScan (http://www.targetscan.org) and miRanda (http://www.microRNA.org) were used to predict potential miR-630 targets. The BCL2L2 (NM_001199839) binding site was predicted to be located at position 995–1002 while TP53RK (NM_033550.3) was predicted to be located at position 455–462 from the transcriptional start site. A 200 bp long BCL2L2 and TP53RK segment were synthesized with either mutant or wild-type seed region and cloned into the psiCHECK-2 vector (Applied Biosystems, USA). Five nucleotides in the seed region were mutated to obtain mutant BCL2L2 and TP53RK 3′-UTR sequences. All cell lines were transfected using Lipofectamine 2000 (Invitrogen, USA). Cells (1×10^5^ cells/well) were transiently transfected with 20 nmol/L miR-630 mimics or control. Then, co-transfection with BCL2L2 and TP53RK-expressing vector were performed. Cells were harvested 48 hours after transfection. The Dual-Luciferase Reporter Assay System (Promega, Madison, USA) was used to detect luciferase activities.

Transcription factors (TFs) regulating miR-630 were predicted using Consite (http://asp.ii.uib.no:8090/cgi-bin/CONSITE/consite), Transfac (http://www.gene-regulation.com/pub/databases.html), and DIANA (http://diana.imis.athena-innovation.gr/DianaTools/index.php). Sequences of the 1,000-bp segment of 5′-UTR of miR-630 with the mutant or wild-type seed region were synthesized and cloned into the PGL3-Basic vector (Applied Biosystems, USA). Five nucleotides in the seed region were mutated to obtain the mutant sequence. cAMP response element-binding (CREB) protein coding sequence was cloned into the pcDNA3.1 vector (Applied Biosystems, USA). Cells (1×10^5^ cells/well) transiently transfected with the mutant or wild-type 5′-UTR of miR-630 were seeded. Then, co-transfection with CREB-expressing vector was performed. After 48 hours, cells were harvested and luciferase activity assays were performed.

### Western blot

Bradford DC protein assay (Bio-Rad, USA) was used to measure the concentration of protein lysates. Subsequently, 20–40 μg of protein was resolved on a 12% Bis-Tris polyacrylamide gel, electrotransferred onto nitrocellulose membranes (GE Healthcare, Piscataway, USA) and blocked with 5% nonfat-milk. Protein blots were immunostained overnight with primary antibodies at 4°C and for 1 hour with secondary antibodies at room temperature. Amersham ECL Western Blotting Detection Reagents (GE Healthcare) were used to visualize the protein signal.

### Treatment of cells with 5-aza-2′-deoxycytidine

HCT116 cells were seeded on 6-well plates on day 0, then 5-aza-CdR (5-aza- deoxycytidine, Sigma–Aldrich), which can inhibit DNA methyltransferase, was added to the cells from day 1 to day 3. Cells were then harvested and ethanol was added to fix cells. Expression of miR-630 was analyzed by qRT-PCR.

### Chromatin immunoprecipitation (ChIP) assay

ChIP assay was conducted in the CRC cell line SW480. Anti-CREB antibody (Abcam, USA) with the EpiSeeker ChIP Kit (Abcam, USA) was used as recommended by the manufacturer.

### Statistical analysis

All data are expressed as mean±standard deviation (SD), with each experiment repeated at least three times. The inhibition rate was obtained by following the calculation: (absorbance value of none IR cells—absorbance value of IR cells)/absorbance value of none IR cells. Differences between the two groups were assessed via 2-tailed, paired student’s t-test. SPSS for Windows 20.0 software was used to perform all statistical analyses. Statistical significance was inferred if *p*<0.05.

## Results

### Expression levels of miR-630 were reduced in CRC cells after irradiation

**Fig 1:**
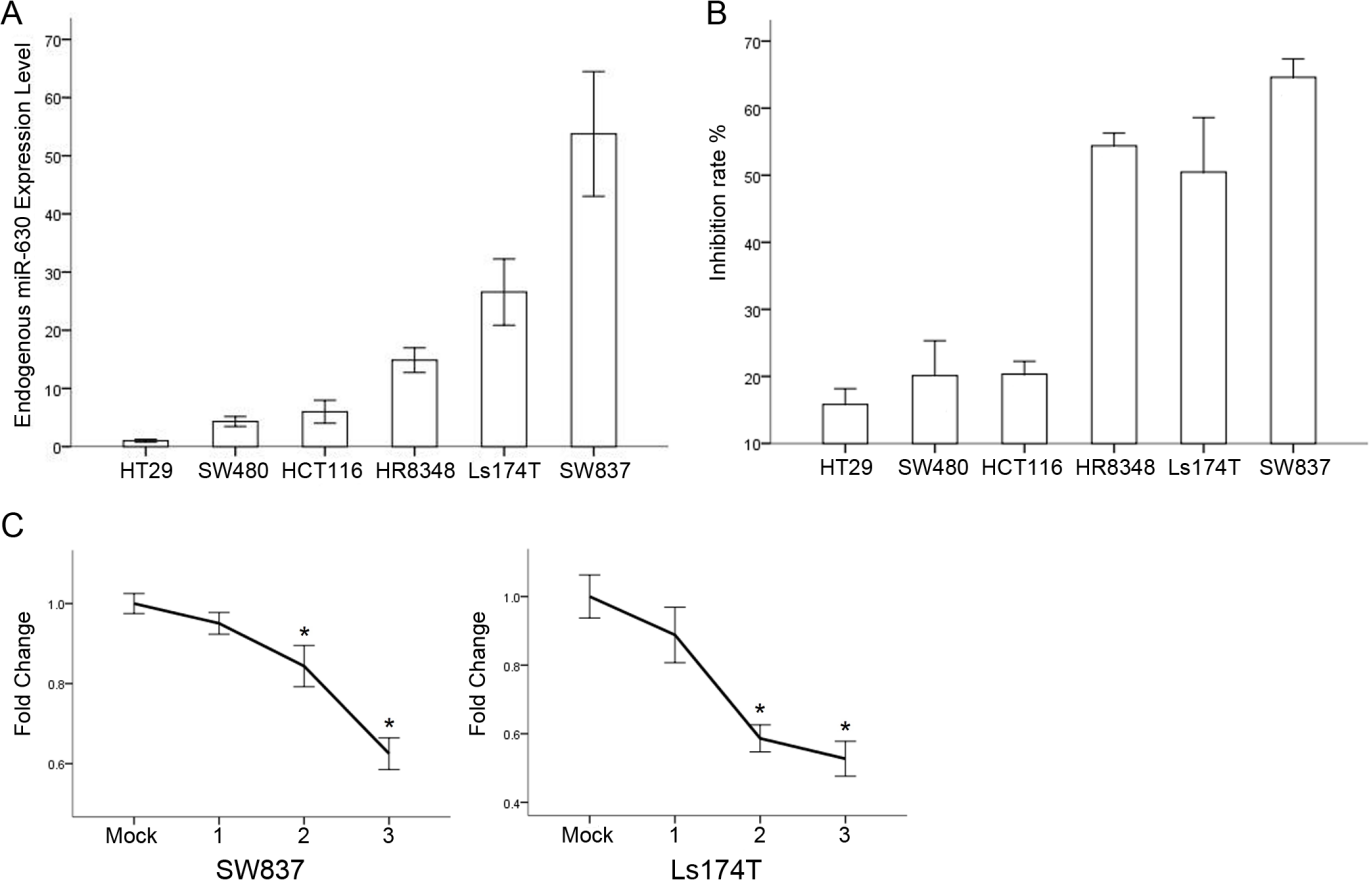
miR-630 expression levels reduced after radiation. (A and B) Endogenous miR-630 expression and IR-induced inhibition rate of serial CRC cell lines (C) miR-630 level decrease after radiation compared with their initial level. Error bars represent the mean of three separate determinations ± standard deviation (SD). Asterisk indicates statistically significant changes: * (P < 0.05), ** (P < 0.01).

The initial levels of miR- 630 and proliferative capacities of serial CRC cell lines were determined to ascertain whether miR-630 can functionally affect ionic radiosensitivity. An apparent inhibitory effect on cell proliferation after ionic radiation was observed in SW837, Ls174T, and HR8348 cells, which showed very high endogenous miR-630 expression; by contrast, weak inhibition rates were observed in HT29, SW480, and HCT116 cells, which exhibited low miR-630 expression (Fig 1A and 1B). Significant positive correlation between miR-630 and radiosensitivity was detected in all six cell lines tested.

Cell lines SW837 and Ls174T, which showed the highest expression levels, were then selected for irradiation. The radiation treatment was repeated thrice, with each treatment consisting of 3 Gy IR. Viable cells were subcultured for 5 d following irradiation, after which miR-630 expression level was examined (Fig 1C). A significant decrease in miR-630 levels was found after irradiation in both cell lines compared with their initial levels.

### Ectopic expression of miR-630 enhanced ionic radiation-induced cytotoxicity in CRC cell lines

**Fig 2:**
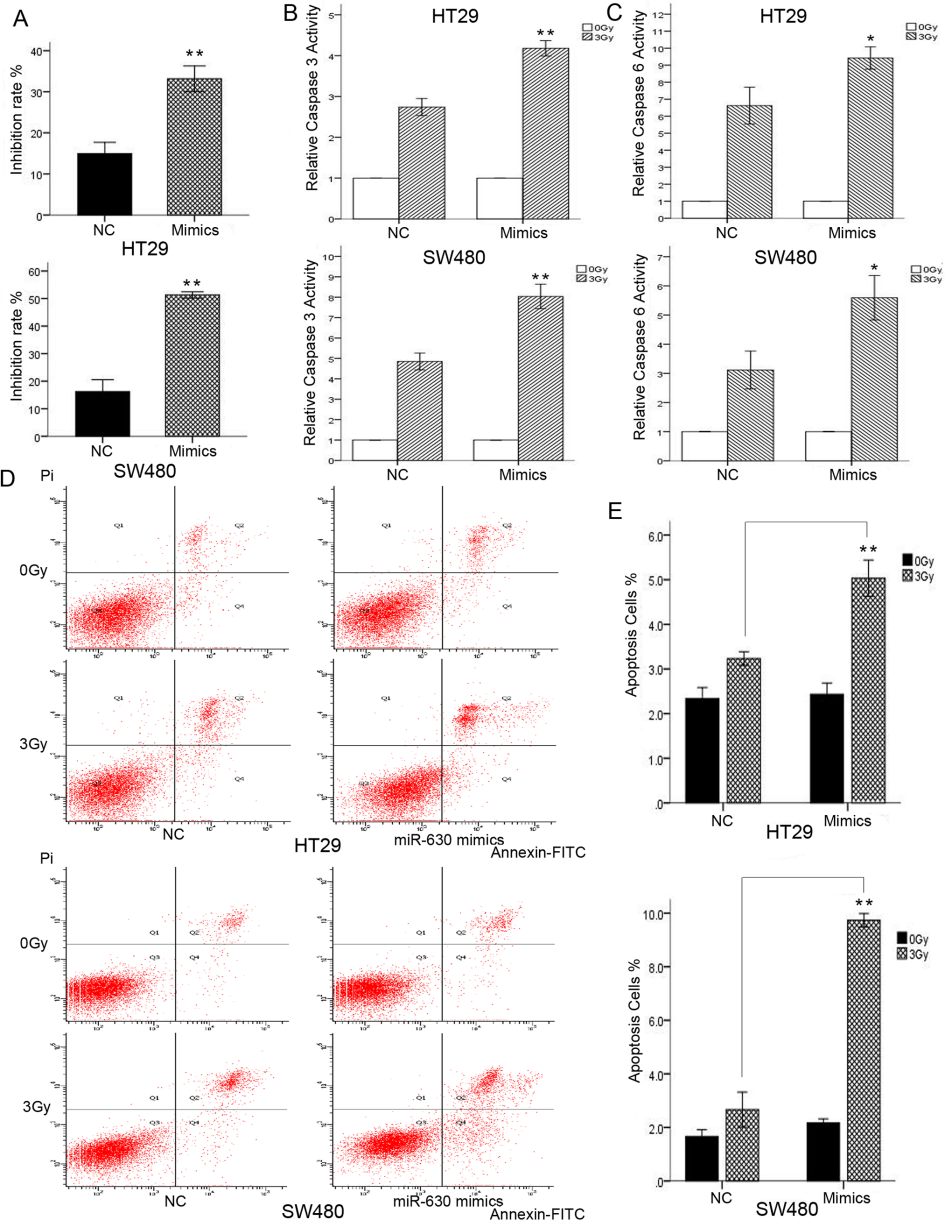
miR-630 overexpression enhanced ionic radiation (IR)-induced cytotoxicity. (A) miR-630 significantly increased IR-induced inhibition rate (B and C) miR-630 strongly increased caspase 3 and caspase 6 activities following IR exposure (D and E) miR-630 significantly increased apoptosis rate after IR exposure. Error bars represent the mean of three separate determinations ± standard deviation (SD). Asterisk indicates statistically significant changes: * (P < 0.05), ** (P < 0.01).

To investigate the role of miR-630 in modulating CRC radiosensitivity, miR-630 was overexpressed in HT29 and SW480 cells by using miR-630 mimics. Subsequently, miR-630-transfected cells were irradiated with 3 Gy IR and subjected to CCK-8 assay along with their control counterparts. Transfection of miR-630 led to significant increase in inhibition of cell proliferation after IR exposure compared with the control groups (Fig 2A) in SW480 and HT29 cells. Caspase 3/6 activities of miR-630-transfected cells and control cells after 24 h with or without 3 Gy IR were also measured.

The caspase 3/6 activities were strongly increased by miR-630 following IR exposure in HT29 and SW480 cells compared with NC cells (Fig 2B and 2C), implying a significant role for miR-630 in IR-mediated apoptosis activation. Additionally, the apoptosis assay illustrated that miR-630 can lead to significantly increased apoptosis rate in the two cells after irradiation (Fig 2D and 2E). Overall, these results suggested that miR-630 sensitizes CRC cells to IR by enhancing IR-induced apoptosis and inhibiting cell survival after IR.

### Inhibition of miR-630 led to decreased IR sensitivity in CRC cell lines

**Fig 3:**
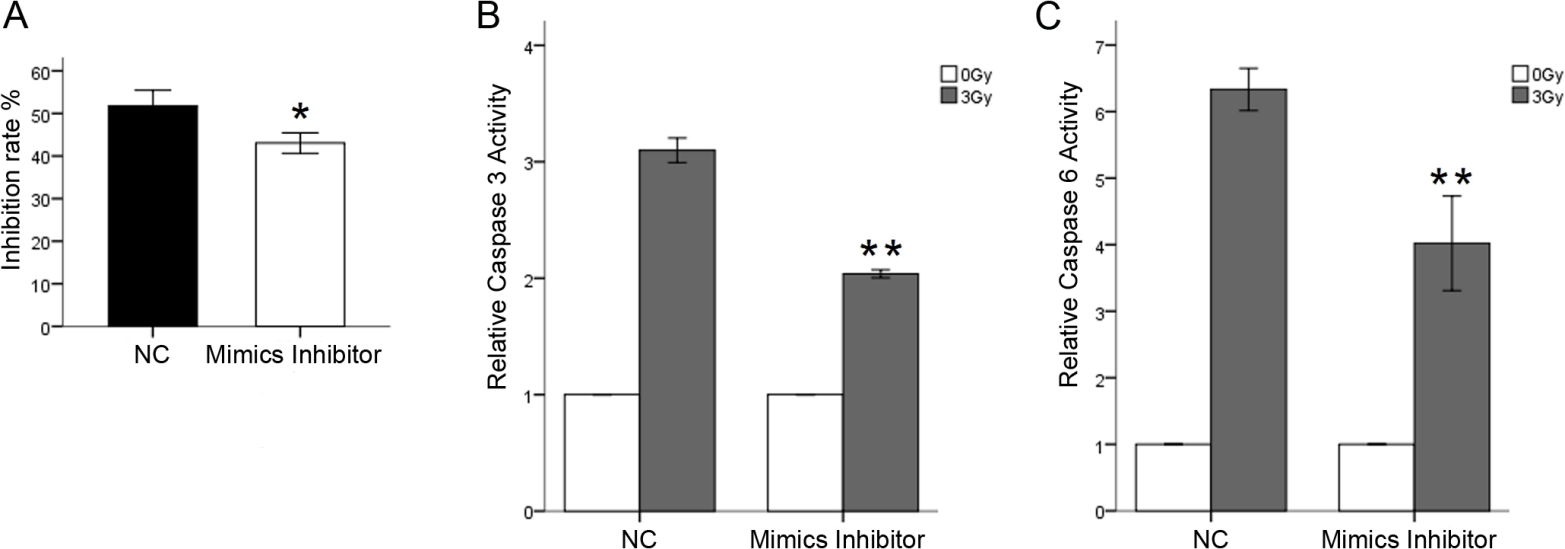
Inhibition of miR-630 led to decreased IR sensitivity. (A) Inhibition of miR-630 significantly decreased IR-induced inhibition rate (B and C) Inhibition of miR-630 strongly decreased caspase 3 and caspase 6 activities following IR exposure. Error bars represent the mean of three separate determinations ± standard deviation (SD). Asterisk indicates statistically significant changes: * (P < 0.05), ** (P < 0.01).

A loss-of-function assay was performed in SW837 cells by using miR-630 inhibitors. Proliferation rate was significantly increased in SW837 cells transfected with the miR-630 inhibitor compared with control SW837 cells treated with IR (Fig 3A). Inhibition of miR-630 also significantly decreased caspase 3/6 activities in SW837 cell lines after IR exposure (Fig 3B and 3C). These results indicated that loss of miR-630 function reduces radiosensitivity of CRC cell lines.

### TP53RK and BCL2L2 are direct targets of miR-630

**Fig 4:**
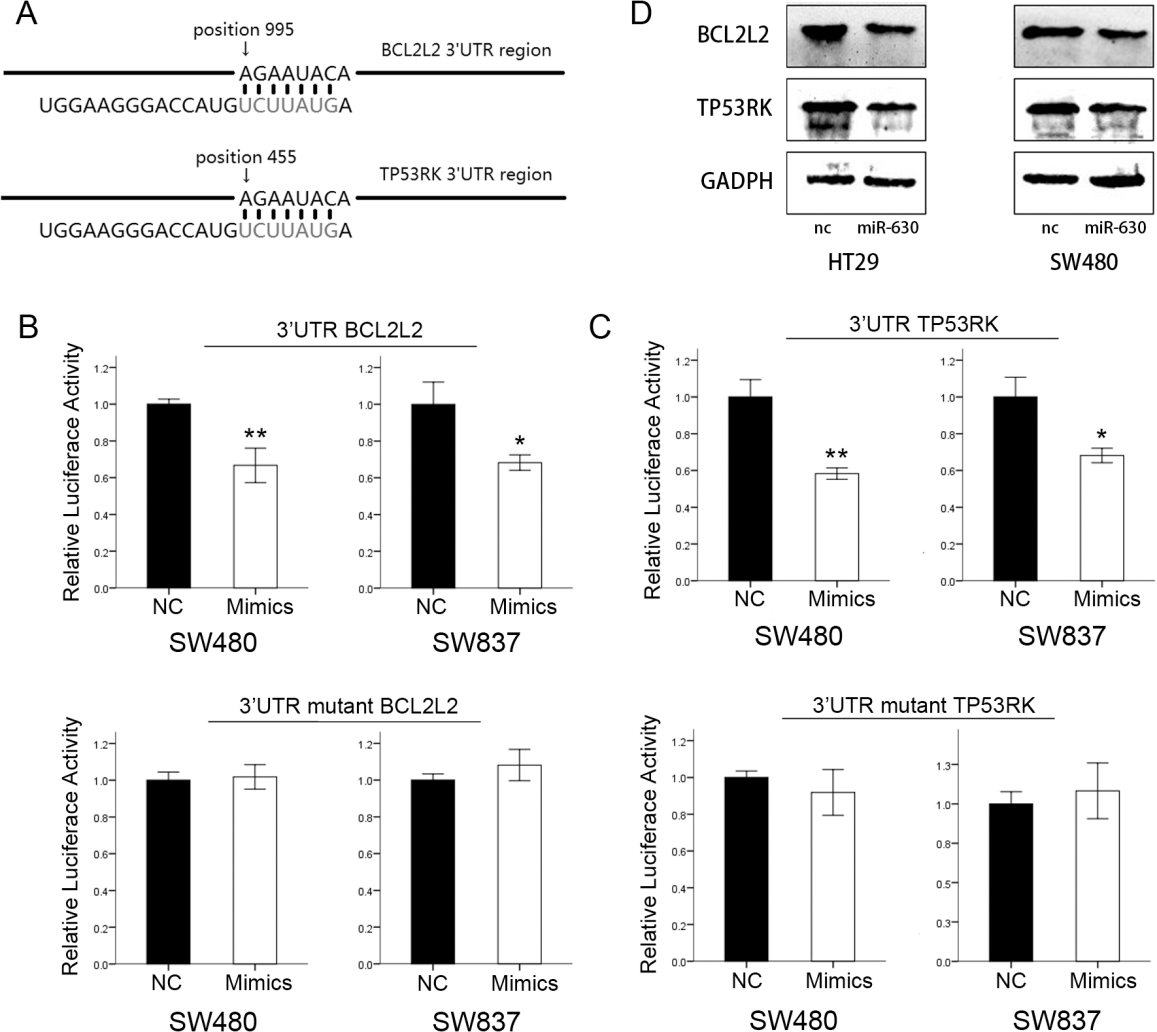
TP53RK and BCL2L2 are direct targets of miR-630. (A) Predicted binding sites of miR-630 in the 3′-UTRs of TP53RK and BCL2L2 (B and C) Fluorescence was significantly reduced in the miR-630 mimics and wild-type dual-luciferase reporter plasmid-transfected group, while it showed no significant change in the miR-630 mimics and mutant dual-luciferase reporter plasmid-transfected group (D) TP53RK and BCL2L2 protein expression levels decreased in miR-630-transfected cells. Error bars represent the mean of three separate determinations ± standard deviation (SD). Asterisk indicates statistically significant changes: * (P < 0.05), ** (P < 0.01).

To explore the molecular mechanisms by which miR-630 increases cellular sensitivity to IR, potential targets of miR-630 were searched in bioinformatics databases via Target-Scan and miRanda. In the 3′-UTR of TP53RK and BCL2L2, potential binding sites of miR-630 were predicted (Fig 4A). To confirm whether miR-630 directly targets the 3′-UTR of these genes, we constructed dual-luciferase reporter plasmids carrying a 200-bp fragment of the mutant or wild-type 3′-UTR sequence, which contained the predicted miR-630 recognition site. A Dual-Luciferase Reporter Assay System was then adopted to determine if miR-630 regulates expression of the predicted candidates in SW480 and SW837 cells. Normalized fluorescence intensity of the reporter was significantly lower in cancer cells co-transfected with miR-630 mimics and wild-type 3′-UTR compared with the control group. In contrast, no significant difference was detected between the control group and the cells co-transfected with miR-630 mimics and mutant 3′-UTR (Fig 4B and 4C).

The above results were also supported by Western blot analyses. Protein expression analysis revealed a sharp decrease in TP53RK and BCL2L2 of miR-630-transfected HT29 and SW480 cells compared with the negative control cells (Fig 4D). Overall, these data demonstrated that miR-630 can directly bind to the 3′-UTR of TP53RK and BCL2L2 to repress gene expression.

**Fig 5:**
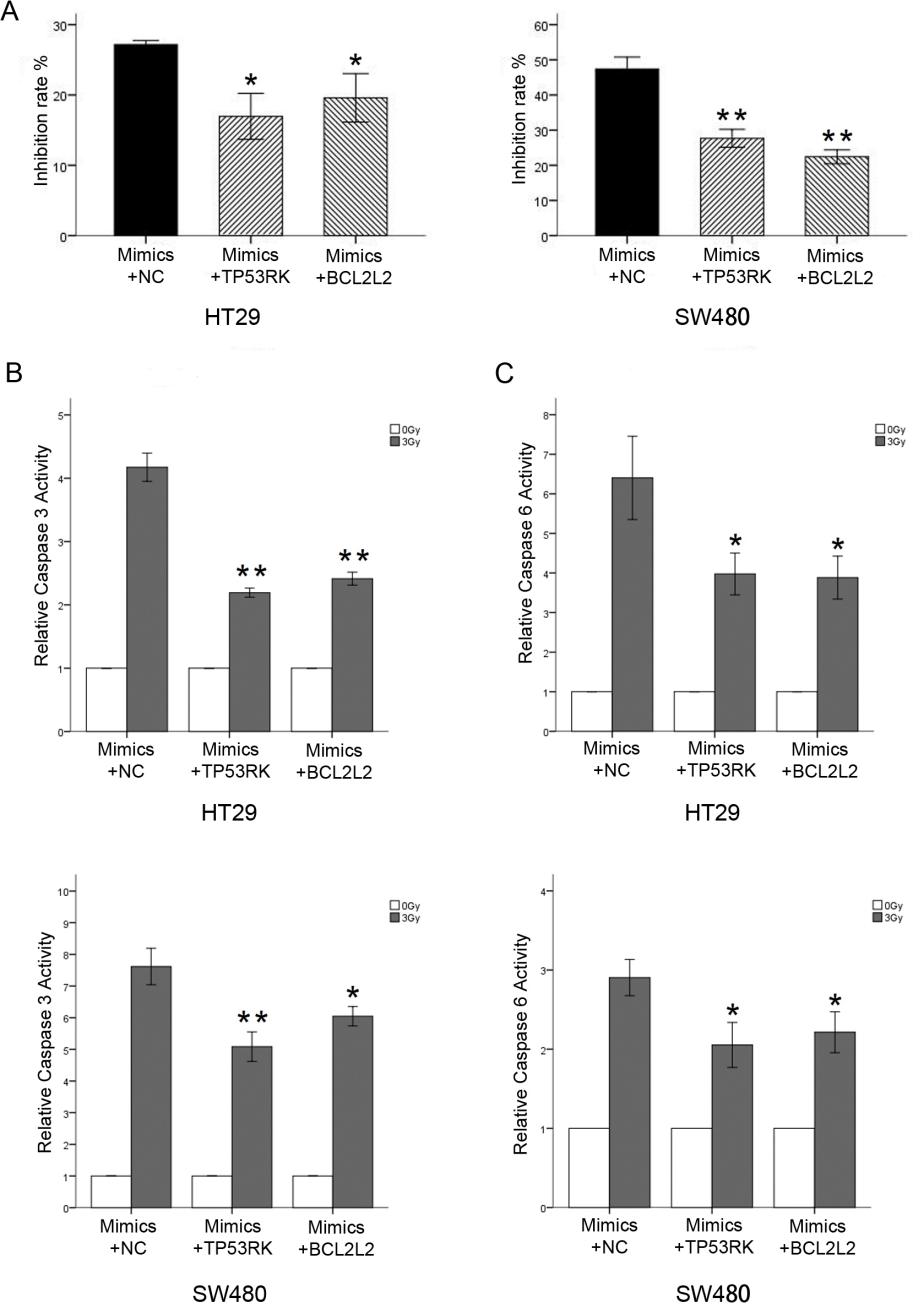
TP53RK and BCL2L2 suppress the effects of miR-630 overexpression. (A) TP53RK or BCL2L2 protein caused a significantly decreased IR-induced inhibition rate in miR-630 transfected cell lines. (B and C) TP53RK or BCL2L2 protein decreased the fold of caspase 3 and caspase 6 activities following IR exposure in miR-630 transfected cell lines. Error bars represent the mean of three separate determinations ± standard deviation (SD). Asterisk indicates statistically significant changes: * (P < 0.05), ** (P < 0.01).

If the effect of miR-630 is specific, the effect of miR-630 overexpression should be suppressed by co-expression of TP53RK and BCL2L2 proteins. The plasmids pcDNA3.1/TP53RK and pcDNA3.1/BCL2L2, which can briefly elevate the levels of TP53RK and BCL2L2, respectively, were co-transfected with miR-630 mimics into HT29 and SW480 cells. Then, caspase3/6 activity assays were performed. CCK-8 assay showed that co-expression of TP53RK or BCL2L2 protein with miR-630 mimics significantly decreased the inhibition rate after IR in both cell lines (Fig 5A). The caspase3/6 fold change post-irradiation in miR-630-transfected SW480 and Ls174T cells decreased significantly after transfection of pcDNA3.1/TP53RK and pcDNA3.1/BCL2L2 (Fig 5B and 5C).

### Cell methylation status and the transcription factor CREB regulate miR-630 expression

**Fig 6:**
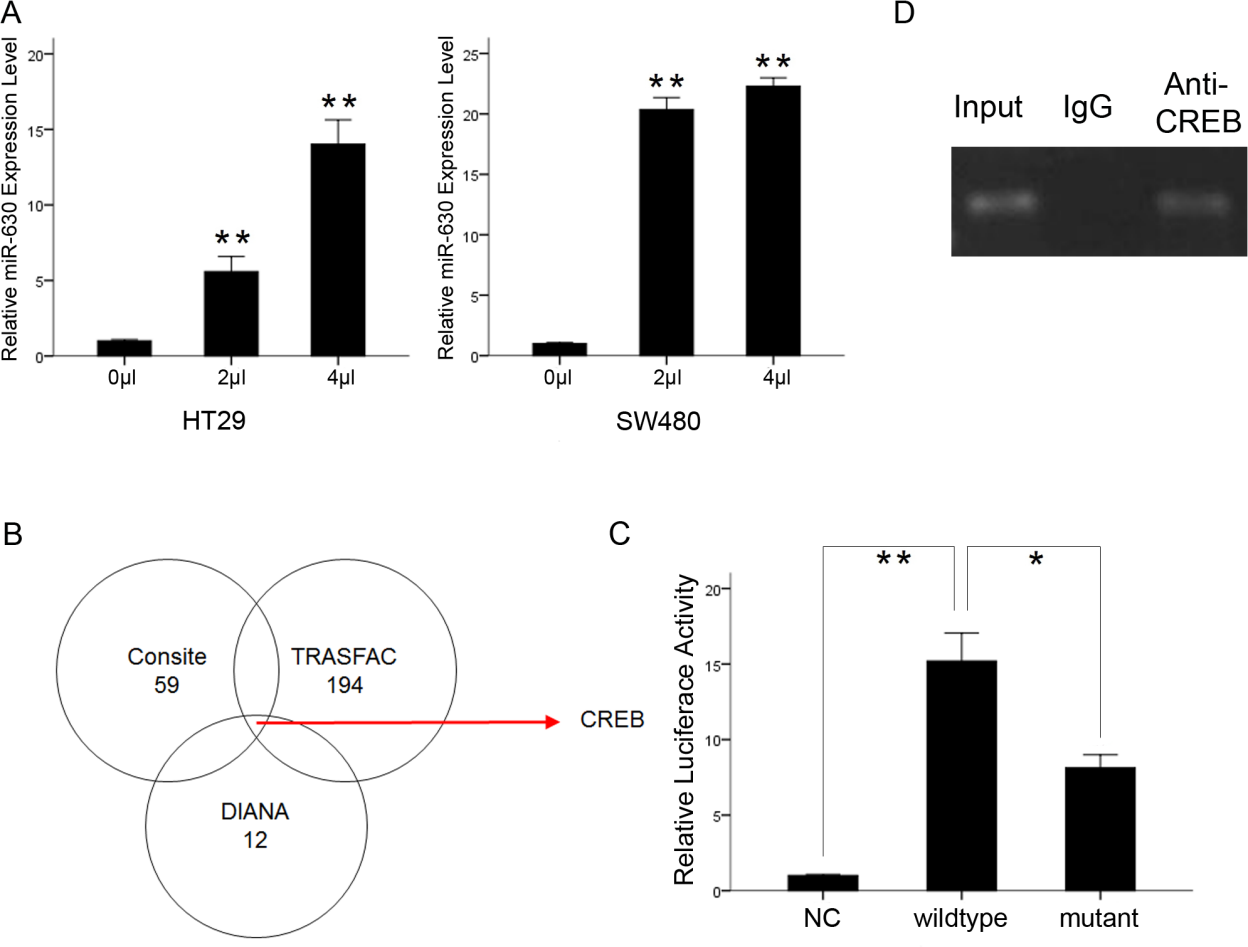
DNA methylation status and transcription factor CREB mediated miR-630 biogenesis. (A) 5-aza-CdR significantly upregulated miR-630 expression (B) CREB was predicted to bind to the 5′-UTR of miR-630 host gene ARIH1 (C) CREB within the ARIH1 promoter region induced luciferase activity and mutation of predicted CREB binding sites reversed its effect (D) ChIP confirmed the binding of CREB to the ARIH1 promoter. Error bars represent the mean of three separate determinations ± standard deviation (SD). Asterisk indicates statistically significant changes: * (P < 0.05), ** (P < 0.01).

DNA methylation can regulate the expression of miRNAs [11, 12]. In this study, miR-630 expression was significantly upregulated by treatment with 5-aza-CdR in HT29 and SW480 cells (Fig 6A). This result indicates that DNA methylation is closely correlated with changes in miR-630 expression during CRC progression.

ARIH1, a protein-coding gene containing the miR-630 gene in its exon, has a large CpG island. This suggests that miR-630 expression depends on regulation of the ARIH1 gene. Three different software programs were used to predict the TFs regulating miR-630. Our data suggested that CREB can bind to the 5′-UTR of ARIH1 (Fig 6B). To determine whether CREB can modulate the level of miR-630, we studied the effect of CREB on mutant and wild-type ARIH1 promoter sequence containing predicted CREB-binding sites. In SW480 cells, we found that CREB-induced luciferase activity was decreased in the group in which the ARIH1 promoter region was mutated (Fig 6C). Furthermore, ChIP assays confirmed that CREB could directly bind to the ARIH1 promoter (Fig 6D).

## Discussion

Despite evidence implicating miR-630 in cancer, only a few studies have explored its function in detail. miR-630 has been identified to induce cell death in pancreatic cancer and to regulate HER-targeting drug sensitivity in HER2-overexpressing breast cancer cells though IGF- 1R [12, 13]. miR-630 inhibits cell growth though CDC7 kinase in human lung cancer cells [14]. Moreover, growth arrest of prostate cancer by gefitinib and luteolin is partly induced by miR-630 [15]. However, the role and expression pattern of miR-630 in radiosensitivity of CRC have not been illustrated to date.

In the present study, we investigated the mechanism by which miR-630 regulates radiosensitivity of CRC cell lines. First, expression of miR-630 and radiosensitivity of six CRC cell lines were detected. The results showed a positive correlation between miR-630 expression and radiosensitivity. In addition, the miR-630 level was found to be significantly decreased after repeated IR, revealing that miR-630 may play an essential role in regulating radiosensitivity.

Next, the possible function of miR-630 in IR response of CRC cell lines was explored. The results showed that overexpression of miR-630 in HT29 and SW480 cells increased cancer cell apoptosis and death *in vitro*. Depletion of miR-630 in SW837 cells had the opposite effect. Thus, these results provide evidence that miR-630 promotes the radiosensitivity of CRC.

The molecular mechanism by which miR-630 modulates CRC radiosensitivity was further investigated. miR-630 was found to negatively regulate the expression of BCL2L2 and TP53RK by directly targeting their 3′-UTRs. BCL2L2, also called BCL-w, belongs to the family of BCL-2 proteins. Overexpression of this protein has been observed in several cancers, including CRC [16, 17]. BCL2L2 also promotes cell survival and reduces apoptosis under apoptotic stress [18, 19]. TP53RK is a kinase that restrains apoptosis after mitotic stress [20]. These findings indicate that miR-630 modulates radiosensitivity via a signaling cascade involving BCL2L2 and TP53RK.

Recently, the E2F1-regulated ribonuclease DROSHA has been found to promote miR-630 biosynthesis in cisplatin-exposed cancer cells [21]. In the current study, luciferase assay results indicated that CREB can bind to the 5′-UTR of ARIH1, which is the host gene of miR-630, and deletion of the CREB-binding sites can abrogate this function. Furthermore, ChIP assay demonstrated that CREB can specifically bind to the promoter region of miR-630, suggesting that CREB may be the TF responsible for initiating miR-630 expression. CREB is a protein that binds to the DNA sequence cAMP response element. CREB mediates target gene transcription via cAMP signal activation [22]. Moreover, CREB is necessary for the proliferation, growth, survival, and differentiation of many cell types [23]. Recent research has demonstrated that CREB-Ser133 phosphorylation results in the suppression of anti-apoptotic genes, thereby inducing cell death [24, 25]. In this study, low DNA methylation also significantly increases miR-630 expression in CRC cell lines.

In summary, our study demonstrates a novel signaling pathway from CREB to BCL2L2 and TP53RK. The present results provide important insights into the mechanisms by which TFs and miRNAs modulate cancer cell radioresistance. Our findings also highlight the therapeutic potential of these molecules in CRC treatment.

## Supporting Information

S1 Data Raw data set is in the “Data.zip”, the original data of this experiment can be obtained from it. (ZIP)
